# A Low-Resolution Infrared Array for Unobtrusive Human Activity Recognition That Preserves Privacy

**DOI:** 10.3390/s24030926

**Published:** 2024-01-31

**Authors:** Nishat Tasnim Newaz, Eisuke Hanada

**Affiliations:** 1 Graduate School of Science and Engineering, Saga University, Saga 8408502, Japan; 22805191@edu.cc.saga-u.ac.jp; 2 Faculty of Science and Engineering, Saga University, Saga 8408502, Japan

**Keywords:** human activity recognition, low-resolution infrared array, thermal detection, interpolation, machine learning

## Abstract

This research uses a low-resolution infrared array sensor to address real-time human activity recognition while prioritizing the preservation of privacy. The proposed system captures thermal pixels that are represented as a human silhouette. With camera and image processing, it is easy to detect human activity, but that reduces privacy. This work proposes a novel human activity recognition system that uses interpolation and mathematical measures that are unobtrusive and do not involve machine learning. The proposed method directly and efficiently recognizes multiple human states in a real-time environment. This work also demonstrates the accuracy of the outcomes for various scenarios using traditional ML approaches. This low-resolution IR array sensor is effective and would be useful for activity recognition in homes and healthcare centers.

## 1. Introduction

Understanding people’s activities is important to their health and for improving their lives. This is especially true in smart buildings that use advanced technology. With the Internet of Things (IoT) with smart technology, we are now able to connect how people move and act, which is changing how we live. For instance, it helps to automatically control things like lights and air conditioning in homes based on what people are doing. It is also useful for keeping an eye on the health of older people by seeing how active they are without invading their privacy directly [1]. The aging of the population requires innovative support systems that prioritize the well-being of elderly individuals while maintaining their autonomy. Monitoring systems designed for this purpose often compromise privacy: creating ethical concerns and hindering widespread acceptance. Addressing these challenges has led to the exploration of non-intrusive, privacy-centric technologies [2]. Activity recognition can be divided into two types: wearable and non-wearable. The preference often leans towards non-wearable sensors among older individuals due to their simplicity and independence. Non-wearable sensors offer a more user-friendly experience because they do not require the user to wear or manage specific gadgets, which aligns better with the comfort and convenience desired by many elderly people. Wearable methods need gadgets like smartphones or sensors such as fitness bands worn by the person. They are good for accurate tracking and mobility but are not great for older people who have to wear these gadgets all the time [3]. Wearable activity recognition is possible with smartphone sensors [4], capacitive sensors [5], acceleration sensors [6], and other such items. Non-wearable methods, on the other hand, use fixed devices like cameras or sensors placed in a room. They are easier to use, but they might not be as accurate and cannot detect everything, which reduces their value during emergencies. Cameras might also invade privacy and not work well in the dark. Sensors placed on the ceiling can recognize activity, but their coverage depends on where they are placed, and they might miss important things like falls. Each method has its pros and cons, such as accuracy, ease of use, and privacy concerns.

Many existing monitoring systems invade the privacy of elderly individuals: raising ethical dilemmas and causing reluctance with their adoption. While sensor-based solutions exist, they often fail to uphold stringent privacy standards, which necessitates the development of innovative approaches that balance effective monitoring with privacy preservation [7].

IR sensors do not capture detailed images or videos like cameras do. Instead, they detect heat signatures to identify movement, ensuring privacy by not capturing identifiable visuals. Because they monitor activity without invading personal space or recording specific details, their anonymity makes IR sensors a more privacy-conscious option, which addresses the ethical concerns related to privacy invasion often associated with camera-based monitoring systems. Infrared (IR) sensors are often preferred over cameras for monitoring elderly persons due to privacy concerns [8].

This work makes the following major contributions:

- We develop and evaluate a system that can recognize human activity in a real-time environment.
- It is a human activity recognition system that does not invade privacy and that is cost-effective.

The rest of the paper is organized as follows: Section 2 illustrates background studies related to this work. The design of the prototype device developed for the system is explained in Section 3. We propose a novel human activity recognition system that is unobtrusive and does not use ML; it uses interpolation and mathematical measures; the methodology of the system is described in Section 4. Traditional ML approaches that can also detect human status are discussed in Section 5. After that, real-world experiments are explained in Section 6. Discussions of the results obtained from the experiments, obtained features, and limitations are given in Section 7. Finally Section 8 is the conclusion and lists possible future work.

## 2. Literature Review

The suggested approach in [9] divides the temperature distribution from the sensor into five basic states: “No event”, “Stopping”, “Walking”, “Sitting”, and “Falling” (emergency). They suggested the use of an infrared array sensor with low resolution for activity recognition. The sensor is appropriate for the surveillance of older individuals based on its general adaptability in low light, expense, dimensions, confidentiality, and availability. They came up with a fall detection technique that uses the sensor to alert people about random falls. The study of [10] introduced a deep-learning-network-based HAR method based on a low-resolution infrared array sensor through which 8 × 8 infrared thermal pictures for identification are gathered from the infrared sensor. The findings showed 98.287% accuracy for distinguishing between everyday behaviors of lying, standing, sitting, walking, and leaving. A low-cost, highly accurate sensing solution is provided by the suggested device-free HAR system for use in privacy-preserving smart home settings. Paper [11] presents an activity detection system based on infrared sensors. Combining wall and ceiling sensor data with CNN and LSTM achieved 97% accuracy. Comparisons with conventional methods validate the effectiveness of the authors’ approach. Their impressive results demonstrate the superiority of this hybrid deep learning model. In the preliminary work of [12], low-resolution infrared data recorded by synchronized GridEYE^®^ sensors are used to investigate human activity recognition with three classification techniques: SVM, RF, and KNN. The findings demonstrate promising recognition performance for both small and large gesture sizes, with accuracy ranging from 71% to 97% across all gesture classes. Random forest highlighted exceptional performance, achieving 100% accuracy in eight gesture classes from the three sensors. Overall, the study had an accuracy of 82.44%, indicating significant potential in the realm of low-resolution image recognition, particularly within healthcare applications. Study [13] reports an infrared monitoring system to spot where people are and what they are doing without disturbing them. The authors used two thermal sensors to gather data from volunteers doing different things and used deep learning to determine the activities from the images. Their method worked better than other techniques, especially when they used both sensors together. They focused on spotting falls and lying down and obtained high scores: around 97% accuracy overall. Study [14] addressed human activity recognition using low-resolution IR sensors in various settings. They introduced a new dataset, Coventry-2018, for use with Infra-ADL2018 analysis. Their innovative noise removal and two main approaches improved classification accuracy. Single-sided sensors use simplified setups, and smaller layouts show better accuracy. Their methods adapted well to different sensor setups and datasets, such as Infra-ADL2018, demonstrating versatility. The researchers of the article [15] proposes an activity recognition approach that employs a low-resolution infrared array sensor to achieve broad adaptability and privacy. The technology has an enormous range of applications and ameliorates the drawbacks of current approaches, such as violations of privacy. To increase precision, this system is equipped with a long short-term memory classifier. The procedure accomplishes the goal of high-precision human motion detection. The researchers from article [16] introduced a smart way to use low-cost infrared sensors that recognize human activities despite changing environments. They designed a system, SCDNN, with parts that work together to better understand activities. By cleverly using data from various places, they achieved 92.12% accuracy at recognizing activities. This method is better and cheaper than other ways to understand activities in different environments. The literature on human activity recognition (HAR) using sensor technologies presents a rich tapestry of research endeavors, each contributing distinct insights and methodologies. The researchers of [17] propose a comprehensive approach by integrating Internet of Things (IoT) devices and thermal imaging for activity recognition in residential spaces. The study emphasizes the significance of context-aware monitoring and demonstrates the efficacy of this fusion for enhancing the precision of human activity detection within home environments. Karayaneva et al. (2023) delve into the realm of AI-enabled healthcare, focusing on the utilization of low-resolution infrared sensor data for human activity recognition. Their work showcases the potential of these sensors in healthcare settings and provides a foundation for advanced patient monitoring systems [14]. Yuan et al. contribute to the field with a focus on interpretability in passive multi-modal sensor fusion for human identification and activity recognition. This study not only advances recognition accuracy but also addresses the critical issue of model interpretability, enhancing the trustworthiness of HAR systems [18]. In a different vein, Yin et al. explore device-free human activity recognition using low-resolution infrared array sensors and long short-term memory (LSTM) neural networks. Their approach allows for flexibility in deployment by eliminating the need for wearable devices, showcasing the potential for widespread application [10]. Lemieux and Noumeir introduce a hierarchical learning approach for human action recognition, adding a layer of sophistication to the analysis of complex activities [19]. Finally, Luo et al. contribute to the simultaneous tracking and recognition domain by employing pyroelectric infrared sensors for indoor applications [9,10,11,20] to showcase the efficacy of low-resolution infrared sensors in diverse contexts: from fall detection to healthcare applications. The integration of deep learning, hybrid models, and innovative datasets, as demonstrated by various researchers, contributes to the ongoing evolution of accurate and versatile HAR systems.

The proposed work incorporates interpolation and mathematical measures strategically designed to enhance the accuracy of human activity recognition and overcome the limitations of previous works. While avoiding the complexities associated with machine learning, our proposed method leverages mathematical modeling to capture and interpret patterns in thermal pixel data. The interpolation techniques employed contribute to a more nuanced understanding of human states, allowing the system to recognize a wider range of activities with improved precision. The unobtrusive nature of our approach, which avoids detailed image processing and machine learning, contributes to privacy preservation. This is a key advantage, especially in sensitive environments like homes and healthcare centers, where maintaining privacy is of utmost importance. By focusing on direct and efficient recognition without the need for extensive computational resources, our work aims to strike a balance between accuracy and simplicity, overcoming the potential limitations associated with the reduced recognition accuracy of traditional ML approaches.

## 3. Prototype Design

**Figure 1:**
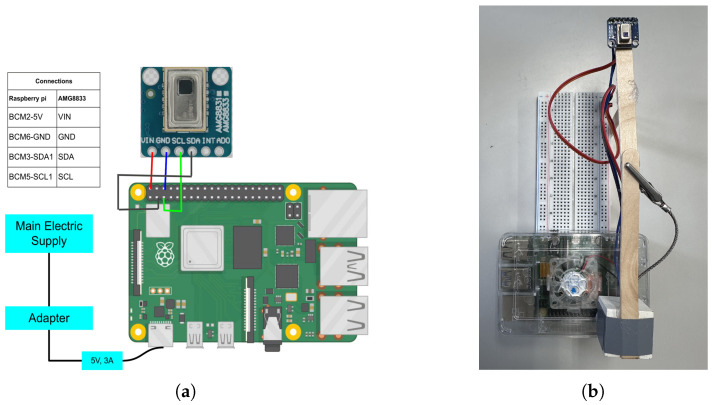
Prototype of our low-resolution IR array HAR device. (**a**) Circuit diagram. (**b**) Real-world prototype (length = 18 cm; width = 9.5 cm).

The prototype of the device is made with a Raspberry Pi (UK), an AMG8833 IR array sensor (Panasonic, Osaka, Japan), a breadboard, a wire, and a power supply. The AMG8833 is an IR sensor array that can be used as a low-resolution thermal imaging sensor and stands out for its exceptional performance. It can sense temperatures ranging from −20 °C to 80 °C and has an 8 × 8 pixel array for thermal mapping. It can measure temperature with 0.5 °C accuracy with the thermistor integrated and with 1.0 °C accuracy using the thermal pixels from the highest distance of 70 cm. It is known for the high accuracy of its temperature readings, energy-efficient design, and user-friendly communication interface. The prototype is described in Figure 1. The AMG8833 is connected to a Raspberry Pi 4 on a table, with the prototype positioned horizontally.

## 4. Proposed Unobtrusive Method

### 4.1. Hypothesis

Low-resolution IR array sensors can map temperature values in a two-dimensional array that can construct a silhouette of the human body if it is efficiently interpolated. The interpolated silhouette will not interfere with privacy. With the average temperature from the interpolated silhouette, we can detect human positions without ML in a real-time environment.

### 4.2. IR Array Mapping and Interpolation

The AMG8833 sensor has an 8×8 array of IR sensors, which allows it to show the temperatures with 64 pixels, with which we can draw a temperature map that represents the silhouette of a human figure. This will be used in later calculations to show a person’s current state and movements. Because 64 pixels is too low resolution to make a full shape of the human body and recognize its state, we used the interpolation method. Overly high interpolation can invade privacy and create pressure on the cost of processing units. Factors related to optimum interpolation are discussed in the results section.

Image interpolation is a technique used to estimate pixel values at non-integer coordinates within an image. It is commonly employed in image processing to resize images, rotate them, or perform other geometric transformations. There are various interpolation methods, but one of the simplest and most used is bilinear interpolation. Though we are not working with image processing, we use bilinear interpolation here to recognize the thermal pixel values on a broad map.

Bilinear interpolation assumes that the pixel intensity varies linearly between neighboring pixel values. Let us denote the four nearest pixel values as $I_{00},I_{01},I_{10},$ and $I_{11},$ corresponding to the pixel values at coordinates $\left(x_{0},y_{0}\right),\left(x_{0},y_{1}\right),\left(x_{1},y_{0}\right),$ and $\left(x_{1},y_{1}\right)$, respectively.

The interpolated value I(x,y) at coordinates (x,y) can be expressed as:

(1) $$\begin{array}{c} I\left(x,y\right)=\left(1-f\right)\left(1-g\right)I_{00}+f\left(1-g\right)I_{10}+\left(1-f\right)gI_{01}+fgI_{11} \end{array}$$

Here,

(x,y) are the target coordinates where you want to estimate the pixel value;

$x_{0},x_{1},y_{0},y_{1}$ are the coordinates of the four nearest pixels.

The normalized distance between *x* and $x_{0}$, which is *f*, is relative to the interval $\left[x_{0},x_{1}\right]$ and can be calculated as follows:

(2) $$\begin{array}{c} f=\frac{x-x_{0}}{x_{1}-x_{0}} \end{array}$$

In same way, the normalized distance between *y* and $y_{0}$, which is *g*, is relative to the interval $\left[y_{0},y_{1}\right]$ and can be calculated as:

(3) $$\begin{array}{c} g=\frac{y-y_{0}}{y_{1}-y_{0}} \end{array}$$

This formula essentially computes a weighted average of the pixel values in the neighborhood of the target coordinates, with the weights determined by the distances *f* and *g*. The closer the target coordinates are to a specific pixel, the higher the weight assigned to that pixel’s intensity in the interpolation.

### 4.3. Proposed Unobtrusive Detection of Human Position with an IR Array without ML

Unobtrusive detection means real-time detection of human activity without applying machine learning approaches. Without machine learning, the detection of human states and their changes is quite challenging and mathematical. However, it is possible. Let us consider the following values:

- px= pixels to read (in our condition, it is 8×8=64);
- ipf= interpolation factor (from multiple tests, ipf=10 is optimal because this value gives the most efficient results with real-time output);
- ipx= total pixels after interpolation (so, ipx=px×ipf);
- t= temperature of the environment;
- pt(n)= temperature value of the *n*-th pixel (here, 0<=n<=px);
- $A_{1}=$ the average temperature of the object in front of the IR array sensor before interpolation;
- $A_{2}=$ the average temperature of the object in front of the IR array sensor after interpolation;
- z(i)= temperature value of the *n*-th pixel after interpolation (here, 0<=i<=px×ipf);
- d= distance between sensor and object;
- df= difference factor, which increases if the distance between the sensor and the object increases, and vice versa.

So, the value of $A_{1}$ can be calculated as follows:

(4) $$\begin{array}{c} A_{1}=\frac{\sum_{n=0}^{px}pt\left(n\right)}{px} \end{array}$$

Now, we can calculate the difference factor df as follows:

(5) $$\begin{array}{c} df=\frac{A_{1}}{t} \end{array}$$

The value of $A_{2}$ can be calculated as follows:

(6) $$\begin{array}{c} A_{2}=\frac{\sum_{i=0}^{ipx}z\left(i\right)}{ipx} \end{array}$$

Now, with our proposed method, we can detect the following states:

1. If $A_{2}>\left(px\times t\times df\right)$: the person is standing;
2. If $A_{2}<\left(px\times t\times df\right)$: the person is sitting;
3. If df is increasing, the object is coming toward the sensor, and if df is decreasing, the object is going away from the sensor.

### 4.4. Algorithm

By following Algorithm 1, we achieved real-time detection of human positions using an IR array and interpolation to visualize them, and we then save the data.

#### Rationale and Comprehensive Explanation

The proposed unobtrusive method relies on the use of an AMG8833 sensor, which has an 8 × 8 array of IR sensors. The key technique employed is image interpolation: specifically, bilinear interpolation. The algorithm for bilinear interpolation is detailed in Equations (1)–(3). Bilinear interpolation was chosen due to its simplicity and efficiency. It assumes linear variation between neighboring pixel values, making it suitable for creating a smooth temperature map of the human silhouette. The choice of an interpolation factor of 10 was determined through multiple tests to balance efficiency with real-time output.

The unobtrusive detection method involves calculating average temperatures before and after interpolation as well as a difference factor (df) based on the environment’s temperature. This method follows Equations (4)–(6). The difference factor (df) is crucial for adapting to changes in the environment’s temperature in order to ensure robustness in detection. The conditions for standing, sitting, and detecting movement are based on the relation between the average interpolated temperature and the product of the original resolution, environmental temperature, and df. These conditions are derived from empirical observations and testing. The detection rationale with mathematical conditions is as written in Section 4.3.

The provided Algorithm 1 outlines the step-by-step implementation of the unobtrusive detection method. It involves initializing the sensor, reading temperature pixel data, mapping pixel data, updating pixels with interpolation, plotting temperature values, and saving raw temperature pixel values to CSV files. The real-time detection loop continuously reads sensor data, calculates average temperatures, and determines the human state based on predefined conditions. The implementation uses the provided mathematical formulas and logical conditions to achieve real-time detection without relying on machine learning. The use of a continuous loop ensures the system’s responsiveness to changes in the environment. The proposed method leverages image interpolation and mathematical calculations to achieve unobtrusive, real-time detection of human positions without the need for machine learning. The choice of bilinear interpolation and the carefully defined conditions for detection states showcase the innovation and effectiveness of the approach. The provided algorithm details the step-by-step process for implementing the proposed method to ensure a comprehensive understanding of its technical aspects.
**Algorithm 1** Unobtrusive detection of human position with IR array interpolation without ML 1:Initialization of libraries and sensor 2:Read data with i2c port 68 or 69 from AMG8833 3:Read 64 pixels of temperature 4:Set figure size and plot the temperature values of the pixels 5:Set color and plot different temperatures using different colors 6:Start process for the original resolution to interpolation 7:original pix.res = (8,8) set to xx,yy 8:new image: zz = np.zeros(pix.res) set to grid.x,grid.y 9:multiplier for interpolation: pix.mult = ipf10:**procedure** interp(px)11:      apply interpolation with interpolation factor ipf12:      return interpolated image z(n)13:**end procedure**14:**for** read temperature pixel data px **do**15:      read environment data with thermistor16:      map the original pixel data17:      update the original pixel to interpolation with procedure INTERP()18:      print the pixel values with matplot19:      Save the raw temperature pixelValues data to CSV files20:**end for**21:**while** True **do**22:      status,pixels = sensor.readTemp(pixToRead)23:      **if** status is null **then**24:            Continue25:      **end if**26:      t = sensor.readThermistor()27:      avg1 = sum(pixels)/len(pixels)28:      df = avg1/t29:      currentValue = df30:      change = currentValue − previousValue31:      **if** currentValue > previousValue **then**32:            Coming Forward33:      **else**34:            Going Backward35:      **end if**36:      previousValue = currentValue37:      newZ = interp(np.reshape(pixels,pixRes)) avg2 = (sum(sum(newZ))/len(newZ)) × df38:      **if** avg2 > t × pixToRead × df **then**39:            standing40:      **else**41:            sitting42:      **end if**43:      sleep(0.625)44:except KeyboardInterrupt: exit45:**end while**

## 5. Traditional ML Approaches for Human State Detection

The pixel mapping of low-resolution IR can detect human position with ML without interpolation. Prediction of human position with ML is quite easier than the proposed direct method, so we need to compare our direct method results with those of ML methods. All the pixel-wise data are saved into a CSV file that can be used in machine learning. We also applied machine learning to recognize the states. We had greater success at recognizing the “Sitting” and “Standing” states with ML applied to our collected data. We applied ML to the raw data without interpolation, so our dataset had 64 columns with an infinite number of rows. We collected all the pixel values from AMG8833 and saved them in a CSV file with their recorded times. We tried multiple ML algorithms and chose the four most suitable algorithms for our datasets as follows:

1. K-nearest neighbors (KNN) is an instance-based classification algorithm that classifies a data point based on the majority class of its k-nearest neighbors. Given a dataset *D* with feature vectors $X_{i}$ and corresponding class labels $y_{i}$, the classification decision for a new data point $X_{\mathrm{new}}$ involves calculating the euclidean distance between $X_{\mathrm{new}}$ and every other point in *D*. Mathematically, the euclidean distance is given by
  $$d\left(X_{i},X_{\mathrm{new}}\right)=\sqrt{\sum_{j=1}^{p}{\left(x_{ij}-x_{\mathrm{new},j}\right)}^{2}}$$
  The class $y_{\mathrm{new}}$ for $X_{\mathrm{new}}$ is determined by the majority class among its k-nearest neighbors [21].
2. Support vector machines (SVMs) aim to find a hyperplane that maximizes the margin between different classes in a high-dimensional space. Given training data $\left(X_{i},y_{i}\right)$, where $X_{i}$ represents feature vectors and $y_{i}$ denotes class labels, SVM solves an optimization problem to find hyperplane parameters β such that $y_{i}\left(\beta \cdot X_{i}+b\right)\geq 1$ for support vectors. Mathematically, the optimization problem is formulated as minimizing $\frac{1}{2}{∥\beta ∥}^{2}$ subject to
  $$y_{i}\left(\beta \cdot X_{i}+b\right)\geq 1$$
  The regularization parameter controls overfitting, and the optimal hyperplane is determined by the support vectors [22].
3. Random forest (RF) is an ensemble learning algorithm that constructs multiple decision trees and combines their predictions. Each decision tree is built on a bootstrap sample of the training data, and at each split, a random subset of features is considered. The final prediction is determined by aggregating individual tree predictions: often through majority voting or averaging. Mathematically, let $T_{i}$ represent the *i*-th tree and $P_{i}\left(y|X\right)$ be the prediction of tree $T_{i}$. The ensemble prediction is given by
  $$P_{\mathrm{ensemble}}\left(y|X\right)=\frac{1}{N}\sum_{i=1}^{N}P_{i}\left(y|X\right)$$
  where *N* is the number of trees [23].
4. Naive Bayes is a probabilistic classification algorithm based on Bayes’ theorem. Given class labels $C_{k}$ and feature vectors $X_{i}$, the posterior probability of class $C_{k}$ given the features is calculated as
  $$P\left(C_{k}|X_{i}\right)=\frac{P\left(C_{k}\right)\cdot P\left(X_{i}|C_{k}\right)}{P(X_{i})}$$
  The independence assumption among features simplifies the likelihood term to
  $$P\left(X_{i}|C_{k}\right)=\prod_{j=1}^{p}P\left(x_{ij}|C_{k}\right)$$
  Naive Bayes classifies a data point by selecting the class with the highest posterior probability. The algorithm is computationally efficient and particularly effective at text classification and other applications where feature independence assumptions hold [24].

## 6. Experiments

### 6.1. Estimation of the Optimal Interpolation Factor for the Proposed Direct Method without ML

We first experimented using the method shown in Section 4.3 to recognize human activity with a direct method and interpolation. In this experiment, we considered the following conditions:

1. Process: Algorithm 1;
2. Subject: One person;
3. Interpolation factor: 1 to 50;
4. Each state tested 100 times.

**Figure 2:**
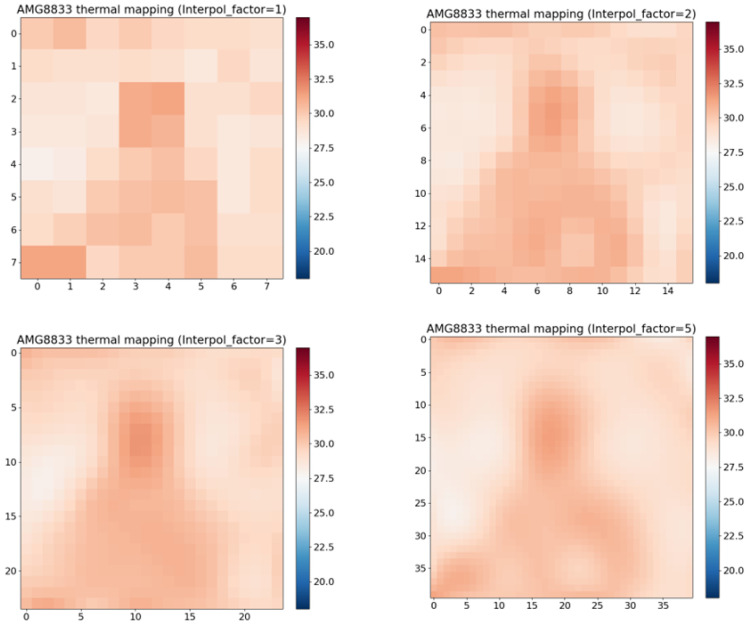
Results of interpolation: interpolation factors are 1 (**Top-Left**), 2 (**Top-Right**), 3 (**Bottom-Left**), and 5 (**Bottom-Right**).

The visualizations of interpolation with various interpolation factors (1, 2, 3, 5) are displayed in Figure 2. If the interpolation factor is 1, that means there is no interpolation. Thus, the top-left figure is without interpolation. We can see that as we increase interpolation, the image becomes clearer.

**Figure 3:**
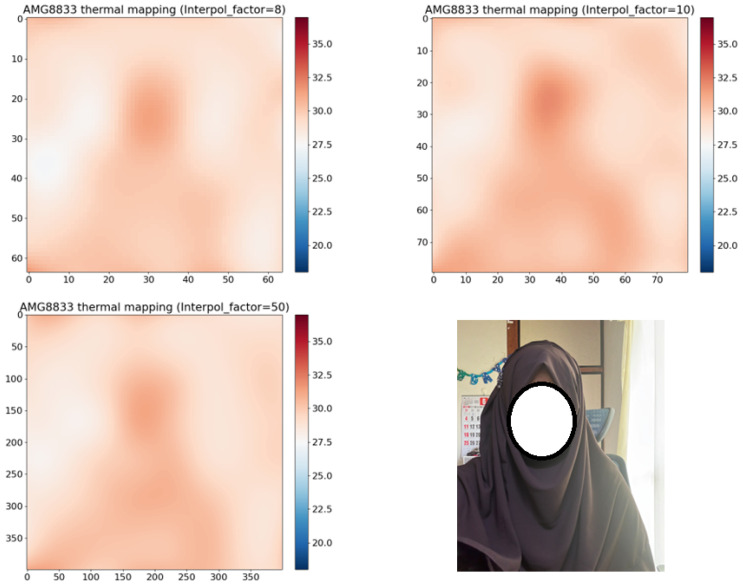
Results of interpolation: interpolation factors are 8 (**Top-Left**), 10 (**Top-Right**), 50 (**Bottom-Left**), and the real image (**Bottom-Right**).

When compared to the original image in Figure 3, interpolation 10 produces a better outcome. The pixels in interpolation 50 are so smooth that they become difficult to discern. Furthermore, the 8×8 pixels become 400 × 400 if the interpolation is set to 50, which can put stress on the Raspberry Pi and complicate the machine-learning method.

When multiple interpolations were used to detect human position states, 252 multiple interpolations of “Standing Still” gave various accuracies for 100 times of testing, as shown in Table 1.

From Table 1 and Figure 3, we can see that interpolation 10 gives the best results. The proposed method worked very successfully. It was able to detect the four states (Sitting Still, Standing Still, Coming toward the sensor, and Going away from the sensor) with the success rates shown in Table 2.

### 6.2. Investigating the Limits of Measurement across Varied Environments

**Figure 4:**
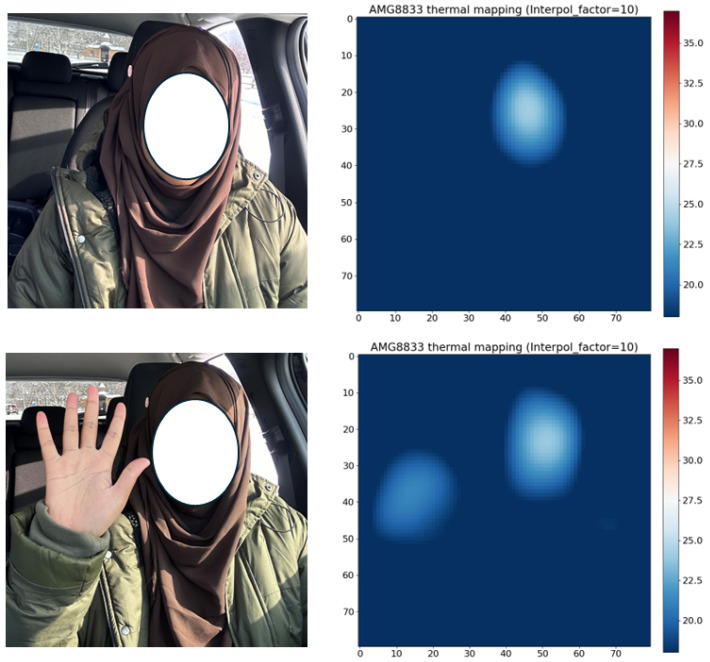
Detection at Lake Toya, Hokkaido. The temperature was −6 degrees centigrade outside and nearly 0 degrees centigrade inside the car. Real image (**left**), Interpolation factor is 10 comparing real image (**right**).

The infrared array sensor is temperature sensitive, so we needed to test the device in different weather conditions. We first collected data in Saga, Japan. When the data were collected, the temperature was 18 degrees centigrade on average. The images shown in Figure 3 were taken at this time. We then collected data at Lake Toya, Hokkaido, at a time when the temperature around the lake was −6 degrees centigrade. The sensor did not work at all for outdoor collection, but it worked well inside the car, where it was 0 degrees centigrade. Figure 4 shows interpolation 10 compared with the real image.

If the person is close to the device, the device can detect only the face. If the person puts a hand up, the device can detect the hand but not detect the whole body because the temperature is very low. If the person were to stand too far from the device at −6 degrees centigrade, it would not detect anything. The device is only useful for indoor detection when the temperature is greater than 0 degrees. The device cannot detect the human body outdoors if the temperature is less than 0 degrees because the temperature difference between the device and the human is very low.

### 6.3. Comparison of Recognition Rates among AI Methods

With our device, we can collect data and save them as a CSV file by following Algorithm 1. We applied various kinds of classified machine learning algorithms to the data we collected. The dataset we considered has 752 rows. Among the various ML algorithms applied, the four previously mentioned were finally used. Their accuracies are shown in Table 3. With the machine learning approach, we can obtain good results without interpolation. However, the process requires more time and processing ability. At a minimum, a Raspberry Pi processing unit is required to process these data, which takes more time than the previously discussed direct approach. Similar results were obtained with the dataset of the authors of [25]. We tested our method with their dataset and got similar results. Here, we consider the following conditions:

1. Processes: ML algorithms mentioned in Section 5.
2. Subject: One person.
3. Training test data for “Standing Still” and “Sitting Still”: The dataset has 752 rows and 65 columns. This dataset was split before training and testing. The training dataset has 602 rows, and the test dataset has 150 rows. The test data were not in the training dataset.
4. Training test data for “SwingFB” and “SwingLR”: The dataset has 1542 rows and 65 columns. This dataset was split before training and testing. The training dataset has 1234 rows and the testing dataset has 308 rows. The testing data were not in the training dataset.
5. Criteria for correctness: We collected data for each state separately. All of the state datasets were then merged into a single dataset with an extra column for their results. The combined dataset was then split into training and testing datasets containing the data for every state. When the training was done with the training dataset, we tested with the testing dataset. Finally, we compared how many results matched with the actual results saved in the extra column.

The accuracy of the results with all the ML methods is shown in Table 3.

## 7. Comprehensive Discussion

### 7.1. Speed Comparison

Table 4 provides a concise speed comparison of the “Standing Still” detection methods, which were all executed on a Raspberry Pi 4B with a 64-bit processor and 4GB RAM. Traditional machine learning algorithms, such as KNN, random forest, SVM, and naive Bayes, demonstrate their performance on this edge device. Notably, the proposed method, which employs a direct mathematical approach, operates efficiently on the Raspberry Pi 4B, showcasing its agility at “Standing Still” detection without the need for a training phase and with minimal computational overhead. The proposed method exhibits a prediction time comparable to or even superior to traditional algorithms, emphasizing its potential suitability for real-time application on resource-constrained edge devices.

### 7.2. Outcomes from Direct Calculation of the Proposed Method

Higher interpolation gives better results, but interpolation higher than 10 makes the scenarios smoother and harder to calculate with low-resolution sensors, which is why interpolation 10 is the best option here. The direct method does not require much processing ability and is very fast to apply in real-time situations. It would be applicable for lower-cost microcontroller-based systems. This method at present can only detect sitting still, standing still, coming towards, and going away. If a person is continuously swinging forward–backward or swinging left–right, the movements cannot be detected by this method. This is not problematic because movement toward or away from the sensors has more practical use than movement swinging forward or backward.

### 7.3. Results Analysis of ML Methods

From the results of Table 3, we can see that with ML, we can successfully predict four states of a person’s movement: sitting still, standing still, swinging forward–backward, and swinging left–right. The RF method shows the best results for sitting still and standing still. On the other hand, SVM shows the best results for swinging forward–backward and swinging left–right. The accuracy summary table reveals distinct performance patterns among the machine learning algorithms employed for human activity recognition. KNN, relying on nearest neighbors, exhibits lower accuracy for “Sitting Still” and “Standing Still”, likely because these stationary activities lack clear clusters in the feature space. However, KNN excels at recognizing swinging forward–backward (SwingFB) and swinging left–right (SwingLR): spatially clustered patterns align well with its proximity-based classification. Random forest (RF) and support vector machine (SVM) showcase consistently high accuracy across all activities, indicating their proficiency at capturing complex decision boundaries and diverse activity patterns. Naive Bayes, while performing reasonably well, lags behind RF and SVM, possibly due to its assumption of feature independence, which may not hold for more intricate activities. In summary, the suitability of each algorithm hinges on the specific nature of the activity, with KNN struggling with the absence of distinct clusters for stationary activities and RF/SVM excelling at capturing nuanced patterns for diverse motions.

A person continuously swinging forward–backward or swinging left–right can be detected by ML methods; however, recognizing a person going away from or toward the sensor is more practical than recognizing swinging forward–backward. This was detected by our direct method but cannot be detected using ML because when going away from or moving toward the sensors, the same distance does not make a big change to the collected static data, which is why we tried to detect moving away and toward the sensors with ML, but this did not provide good results.

### 7.4. Privacy Preservation in the Proposed System

#### 7.4.1. Privacy Concerns in Human Activity Recognition

Privacy preservation is a crucial aspect when dealing with technologies that involve the sensing and analysis of human activities. In the proposed system, the focus on using low-resolution IR array sensors and efficient interpolation contributes to privacy preservation. The key privacy concerns addressed include:

1. Silhouette Representation: By constructing a silhouette of the human body through temperature mapping, the system avoids capturing detailed features or identifying individuals based on visual characteristics. This ensures a higher level of anonymity.
2. No Image-Based Recognition: The system refrains from using image-based recognition techniques and thereby avoids the need for high-resolution sensors or cameras. This minimizes the risk of capturing identifiable visual information and enhances privacy.
3. Non-Intrusive Detection: The term “unobtrusive detection” implies that the system can detect human activity without invasive methods. This means it does not rely on intrusive technologies like facial recognition or detailed body imaging, which reduces the risk of privacy infringement.

#### 7.4.2. Comparative Analysis with Existing Techniques

1. Depth Cameras and Image Recognition:
  - *Privacy Concerns:* Depth cameras, while effective at recognizing human activities, may capture detailed visual information. Image recognition algorithms can be privacy-invasive as they identify individuals based on facial features or body characteristics.
  - *Comparative Privacy:* The proposed system, which uses low-resolution IR sensors and interpolation, provides a higher level of privacy by avoiding detailed visual information.
2. Wi-Fi Sensing:
  - *Privacy Concerns:* Wi-Fi sensing can be used for human activity recognition but raises concerns about tracking individuals based on their Wi-Fi-enabled devices.
  - *Comparative Privacy:* The proposed system, being non-reliant on personal devices, offers a more privacy-preserving approach.
3. Accelerometer-Based Approaches:
  - *Privacy Concerns:* Accelerometer-based techniques can detect movements but might not be sufficient for detailed activity recognition. There is a risk of inferring sensitive information from motion patterns.
  - *Comparative Privacy:* The proposed system, which utilizes temperature mapping, does not rely solely on motion patterns and thus offers a different and potentially less-invasive perspective for activity recognition.
  - *Usability:* Accelerometer-based systems can be less user-friendly as they typically require wearable devices. In contrast, the proposed system eliminates the need for wearables, allowing it to detect activities from a distance without any physical attachment.

### 7.5. Quantitative Metrics and Case Studies

While privacy is challenging to quantify directly, the proposed system can be evaluated using the following metrics and considerations:

1. Resolution vs. Privacy Trade-off: Conduct studies comparing the level of privacy against the resolution of sensors. Higher resolution may provide more detailed information but can compromise privacy. So, the proposed system of working with low-resolution IR is a better choice.
2. Anonymity Preservation: Evaluate the system’s ability to maintain anonymity by analyzing how well it avoids capturing personally identifiable information. The proposed system does not recognize any person’s identity.
3. User Perception Surveys: Collect feedback from users regarding their perception of privacy when interacting with the system. These qualitative data can provide insight into the user’s comfort level. The users involved with the proposed method showed positive feedback.
4. False Positive Rates: Assess the false positive rates for activity recognition. A lower false positive rate indicates that the system is less likely to misinterpret activities, reducing the risk of false accusations or privacy breaches.

The proposed system prioritizes privacy by avoiding high-resolution visual data, focusing on silhouette representation, and employing non-intrusive detection methods. In the future, regular updates and adjustments based on user feedback and evolving privacy standards will be essential for maintaining a high level of privacy in the long term.

### 7.6. Positive Features

The proposed system has multiple positive features as follows:

- It can successfully detect four states of human activity.
- It does not interfere with personal privacy.
- The device is lower cost than the other available human activity recognition systems. It does not require a camera or image processing. The processing unit and sensor are cheaper than other options.
- The device is a convenient size and is completely independent: it does not depend on the internet.
- It will be helpful for monitoring aged people at home.

### 7.7. Limitations

Though this system was generally successful, it has several drawbacks as follows:

- The device is not waterproof.
- The device worked well indoors but showed reduced performance outdoors.
- Though the device worked well at temperatures over 5 degrees centigrade, it failed to work outdoors at temperatures under 0 degrees centigrade.

## 8. Conclusions

In this study, a Raspberry Pi coupled with an AMG8833 IR array sensor was employed for real-time human activity recognition. A novel mathematical approach was introduced that enables the detection of four states: sitting, standing, approaching, and moving away. The direct method we proposed outperformed traditional ML algorithms, including random forest, naive Bayes, SVM, and KNN, demonstrating superior speed, accuracy, and applicability in practical situations. The efficiency of the direct approach not only streamlines computational processes but also outperforms established ML models, emphasizing its practicality and efficacy for real-time applications in smart environments and healthcare settings.

In our future research, we will attempt to detect more states to allow better monitoring of the changing patterns of human movement. Future developments in our system will decrease its limitations, which will allow us to ensure privacy while providing accurate and timely fall detection with low-resolution thermal mapping. The current discussion of ML methods does not delve into the explicit exploration of hyperparameter optimizers and their potential impact on the obtained results. However, it is essential to acknowledge that hyperparameter tuning is a crucial aspect of optimizing the performance of machine learning models. Future work could explore the utilization of hyperparameter optimizers and investigate their potential effects for enhancing the results achieved by the discussed ML methods.

## Figures and Tables

**Table 1:** Accuracy summary for various states with the proposed direct method.

Interpolation	Accuracy of Predicting “Standing Still”
1	10%
2	20.5%
3	40.5%
5	60.5%
10	99.5%
50	88.5%

**Table 2:** Accuracy summary for various states with the proposed direct method.

State	Accuracy of Direct Approach
Sitting Still	98.5%
Standing Still	99.5%
Coming toward the sensor	97.5%
Going away from the sensor	98.5%

**Table 3:** Accuracy summary of the ML algorithms (SwingFB = swing forward and backward; SwingLR = swing left to right).

ML Algorithm	Sitting Still	Standing Still	SwingFB	SwingLR
KNN	56.95%	56.95%	90.67%	90.67%
Random Forest (RF)	98.93%	98.67%	91.45%	91.45%
SVM	98.40%	98.42%	89.11%	89.11%
Naive Bayes	87.76%	88.76%	79.53%	79.53%

**Table 4:** Speed comparison among applied methods for “Standing Still” detection on Raspberry Pi 4B.

Algorithm	Training Time (s)	Prediction Time (s)
KNN	0.0023	0.0837
Random Forest (RF)	0.3908	0.0132
SVM	0.0489	0.0235
Naive Bayes	0.0028	0.0027
Proposed Method	0.0000	0.0024

## Data Availability

The data presented in this study are available on request from the corresponding author.

## Abbreviations

The following abbreviations are used in this manuscript:

IR	Infrared
HAR	Human Activity Recognition
IoT	Internet of Things
ML	Machine Learning
AI	Artificial Intelligence
KNN	K-Nearest Neighbors
SVM	Support Vector Machine
RF	Random Forest
